# Hypertensive Disorders of Pregnancy in a Military Hospital Birth Cohort

**DOI:** 10.1089/whr.2022.0034

**Published:** 2022-08-31

**Authors:** Jimi Francis, Karla Waller, Amber Wilson, Darby Dickton

**Affiliations:** ^1^ Department of Kinesiology, College for Health, Community, and Policy, University of Texas at San Antonio, San Antonio, Texas, USA.; ^2^ Obstetrics, Austin Regional Clinic, Austin, Texas, USA.; ^3^ Indian Health Services, Obstetrics, Women's Health Department, Claremore, Texas, USA.; ^4^ Foundation for Maternal, Infant, and Lactation Knowledge, San Antonio, Texas, USA.

**Keywords:** hypertensive disorders of pregnancy, gestational hypertension, preeclampsia

## Abstract

**Background:**

Hypertensive disorders of pregnancy (HDP) are risk factors for maternal and fetal complications with long-term sequelae for mother and children. HDP are not clearly understood; however, there appears to be a relationship with maternal weight gain. The effects of maternal weight gain and pregnancy outcomes, including HDP, are understudied. Few studies have assessed maternal weight gain in service-connected women and its effects on HDP. This study aimed to evaluate the relationship between blood pressure and birth outcomes in women who delivered their infants at a military hospital.

**Methods:**

This birth cohort study included all patients admitted to a military hospital for delivery over a 12-month period. Data were analyzed for hypertensive disorders, maternal weight gain, delivery type, infant maturity, and infant weight at delivery.

**Results:**

Of the 1,018 participants, 186 were diagnosed with HDP with no statistical difference observed for maternal age. The hypertensive group had higher mean weight gain. More patients in the hypertension group delivered at term with lower mean birth weight. The rate of small-for-gestational age infants was higher in the HDP group (*p* < 0.001).

**Discussion:**

The rate of HDP in this cohort of military members and dependents was 18.3%, which was similar to the 19% rate reported for a southern US hospital, but higher than in other regions of the United States. This evidence indicates that HDP are increasing, and maternal/infant morbidity was affected by hypertension in this study.

## Introduction

As a seemingly preventable disease, hypertension during pregnancy causes an estimated 30,000 deaths each year in the United States^1^ and ∼28,000 deaths in 2019 worldwide,^2^ with over 58% of these maternal deaths thought to be preventable.^3^ Hypertension in pregnancy is defined as a systolic blood pressure >140 mm Hg or diastolic blood pressure >90 mm Hg or diastolic pressure on two occasions at least 4 hours apart, with 20 weeks of gestation used as orientation for clinical management between chronic, gestational hypertension (GH), and preeclampsia (PE) with or without severe features. GH is typically diagnosed when there is elevated blood pressure (140/90 mmHg) after 20 weeks of gestation in the absence of proteinuria (≥300 mg in 24-hour urine collection, protein/creatinine ratio of 0.3, or dipstick reading of 2+).

Women with GH may be diagnosed as preeclamptic with severe features if they experience proteinuria, and other indicators of end-organ damage.^4^ Hypertensive disorders of pregnancy (HDP) include chronic hypertension (CH), GH, PE with and without severe features, CH with super-imposed PE, and eclampsia. In this study, we used the terms CH, GH, PE, and eclampsia. HDP can increase morbidity/mortality, are suspected to be associated with maternal weight, and appear to be increasing in prevalence.^5^

After hemorrhage, hypertension is the most common cause of direct maternal death,^6^ as it can lead to cerebral hemorrhage and convulsions due to dangerously high elevations in blood pressure, leading to eclampsia during pregnancy.^7^ Pregnant women with hypertension have increased complications during pregnancy and deliveries with higher rates of induced labor (IL), preterm delivery, and emergent cesarean section (C-section) delivery than their normopressive counterparts.^8^ Such women are also at risk for future hypertension and cardiovascular disease.^9^

In mothers with HDP, the increase in blood pressure can cause decreased blood flow to the placenta, decreasing the amount of resources available to the fetus,^10^ which in turn can lead to low birth weight or preterm delivery of the infant.^11^ Aside from infant birth complications, infant health can also be at risk, including higher odds of fetal growth restriction,^12^ oligohydramnios, placenta abruption, stillbirth, and the non-reassuring fetal status of inadequate oxygenation noted on antepartum and intrapartum surveillance.^13^ Preterm delivery can lead to further negative health consequences such as breathing problems in the infant due to immature lungs, increased risk of infection, and increased risk of long-term complications such as asthma for the infant.^14^ Long-term implications for the infant born of a hypertensive mother include increased risk for coronary heart disease, type 2 diabetes, and osteoporosis as adults.^15^

The America College of Obstetrics and Gynecology has recommended maternal weight gain in pregnancy with 12.7–18.14 kg for mothers with body mass index (BMI) <18 kg/m^2^ prepregnancy, 11.34–15.88 kg for those with prepregnancy BMI of 18.5–24.9 kg/m^2^, 6.8–11.34 kg for those with BMI of 25–29.9 kg/m^2^, and 5–9.07 kg for those with BMI ≥30 kg/m^2^.^16^ Higher maternal weight gain than the recommended amount is considered excessive maternal weight gain.^17^ Guedes-Martins asserted that HDP is correlated with maternal weight gain^18^ while another study contended that excessive gain during pregnancy is associated with an increasing incidence of maternal and neonatal complications, including HDP.^19^

Although early excessive weight gain during pregnancy has been reportedly associated with an increased risk of developing GH, gain in mid-pregnancy has not been associated with higher blood pressure.^20^ While the timing of weight gain may be a factor, Lewandowska et al. reported that obesity before pregnancy was associated with an increased risk of GH,^21^ indicating that the significance of maternal weight in the development of HDP is uncertain.

With the increased prevalence of obesity,^22^ one would expect that the incidence of hypertension would also increase if there were an association between maternal weight gain during pregnancy and HDP.^23^ The prevalence of CH in the United States has increased over 13-fold, and around the world^2^ HDP occurs between 5.2%^24^ and 8.6%^25^ of overall pregnancy cases, with differences based on category of hypertension. When looking at the most severe HDP, based on US statistics from 2005 to 2014 of delivery hospitalizations involving PE and eclampsia, 53.9% were by C-section, compared with 31.7% of deliveries not involving preeclampsia/eclampsia (PREE).^26^

Women with HDP have a higher incidence of infant health complications, increased birth interventions, and higher weight gain during pregnancy than normotensive (NT) women. It is unknown whether there is an increase in HDP among military members and their dependents delivering at military hospitals in the continental US. The purpose of our study was to evaluate whether the rate of hypertensive disorders among pregnant women obtaining obstetrical services at a military hospital is increasing compared to the general population. The objective of this study was to determine the number of NT women compared to hypertensive women, and any difference between the groups in terms of morbidity, mortality, maternal weight gain, birth interventions, infant maturity, or infant weight at delivery in a military hospital located on the east coast of the United States over a 12-month period.

## Methods

This medical record review was conducted over a 12-month period from June 1, 2017, to May 31, 2018. Data were collected on each mother admitted to the hospital for the delivery of an infant during the 12 months. The variables of maternal weight, birth interventions, diagnosis of hypertension, length of gestation (infant maturity), and infant birth weight were of specific interest. After evaluation of the protocol, the Institutional Review Boards for both the participating hospital and university identified the study as exempt, with no written consent required as any identifying information was de-identified in the data set by the hospital staff collecting the data before receipt by the research team and before data analysis in accordance with human subjects' guidelines.

The study population comprised a convenience sample of all births in a 12-month period. The minimum sample size needed to detect differences between NT and hypertensive participants was calculated by an open epi sample size calculator using a nonprobability convenience method (*n* = 320). However, to increase the size of the sample, the study included all patients who gave birth within the 12-month period. Variables for the specific type of diagnosis of hypertension, length of gestation, birth interventions, length of hospital stay, maternal weight gain, infant maturity, and infant birth weight were evaluated.

Hypertension was diagnosed based on hospital diagnosis codes and grouped as “normotensive” (NT), “chronic hypertension” (CH), “gestational hypertension” (GH), and “preeclampsia/eclampsia.” Analysis was also conducted using the dichotomous variable of a persistently NT status versus any hypertensive disorder (HDP). Descriptive statistics, analysis of variance, and chi-squared tests were used to assess the data with *p* < 0.05.

Maternal morbidity, in this study, was defined as a delivery intervention. The delivery intervention variables were more generalized indicators that delivery was not as expected without isolating only those mothers with severe , as defined by the Centers for Disease Control and Prevention in the Severe Maternal Morbidity Guidance document.^27^

Logistic regression analysis using a forward stepwise method was used to evaluate the relationship between mode of delivery and morbidity using the variables of hypertension, maternal weight gain, artificial rupture of membranes (AROM), IL, and augmented labor (AL). Maternal weight gain was recorded in pounds, but converted to kilograms for consistency as infant birth weight was recorded in grams. As the HDP were categorized as ordinal data, parametric statistics were used to evaluate differences between group means. *t*-Tests, analysis of variance, and crosstab tables were used to evaluate the variables with a significance level set at 95% confidence and *p* < 0.05.

## Results

In this 12-month birth cohort, data were collected from 1,029 mother/infant dyads admitted to the hospital. Eleven participants had diagnosis codes missing from the chart and were excluded from the analysis, leaving 1,018 participants who were included in the analysis. Participants were military members or military dependents as dictated by the population served by the hospital facility and were not differentiated in the charts.

No maternal death was reported in this study. In this cohort, 832 and 186 participants had NT and HDP, respectively. Among the HDP participants, 43 were categorized as CH, 65 as GH, and 78 as PREE. Of those with HDP diagnosis codes, 23% (*n* = 43) had CH, 35% (*n* = 65) had GH, and 42% (*n* = 78) had PREE. The prevalence of CH, GH, and PREE was 4.3%, 6.4%, and 7.7%, respectively.

Of the 1,018 participants, 200 (19.7%) had a C-section with 80.3% delivered vaginally. When grouped, 20.7% of NT, 9.3% of CH, 8.2% of GH, and 21.8% of PREE, respectively, had C-section deliveries. The number of deliveries due to hypertension is shown in Table 1.

**Table 1.** Type of Delivery Mode by Blood Pressure Category

	Normotensive	Chronic hypertension	Gestational hypertension	Preeclampsia	Total
Mode of delivery					
CSEC	172	4	7	17	200
VAG	660	39	58	61	818
Total	832	43	65	78	1,018

CSEC, cesarean section delivery; VAG, vaginal delivery.

**FIG. 1.**
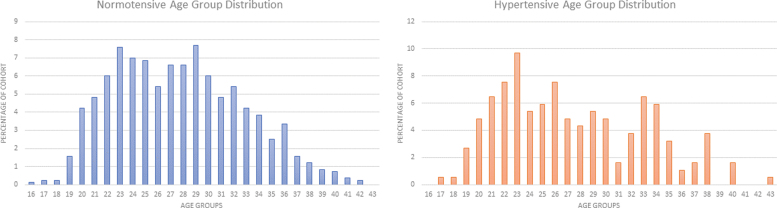
Distribution of maternal age.

The mean age of the participants was 27.6 years of age ranging 16–43 years. The maternal age of the cohort showed a bimodal curve with the two most common being 23 and 29 years. While the NT group was equally dispersed between the two peaks in their bimodal curve, the HDP group predominated in the younger subset of their slightly widened and flattened bimodal curves, as shown in Figure 1.

**FIG. 2.**
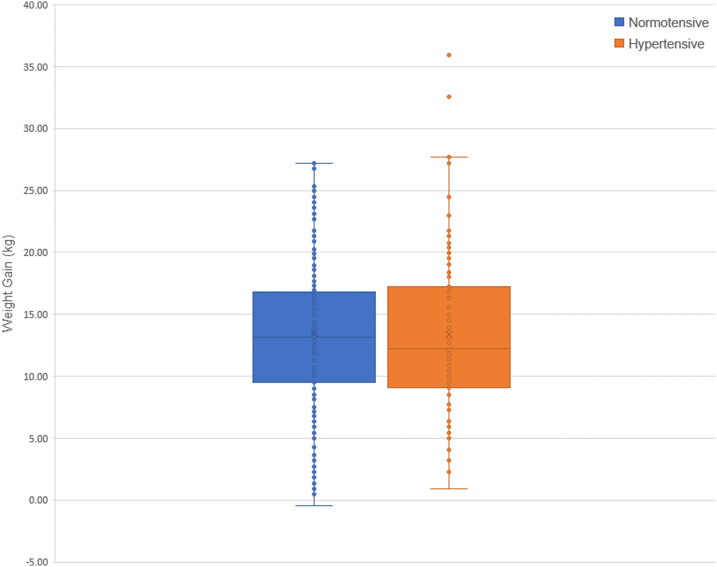
Maternal weight gain was statistically significantly different between NT and HDP groups with *p* = 0. HDP, hypertensive disorder of pregnancy; NT, normotensive.

The mean maternal weight gain was 13.34 in the NT group and 14.39 in the HDP group, which was statistically different (*p* = 0.0001). The distribution, as a box and whisker plot, is shown in Figure 2.

Regression analysis was used to evaluate the predictive value of delivery mode for the variables hypertensive status, maternal weight gain, beta-strep status, AROM, IL, AL, infant sex, maturity, and infant weight. Maternal weight, infant sex, infant maturity, and infant weight were removed from the model because these variables had no predictive value for the final model of significant predictor variables of maternal weight, maternal age, IL, AL, and AROM. The reduced model formed is shown in Table 2.

**Table 2.** Regression Forward Stepwise Analysis

Step summary							
Model		Action	Effect(s)	Model fitting criteria	Effect selection tests		
				−2 Log likelihood	Chi-square^a,b^	df	Sig.
Step 0	0	Entered	Intercept, beta-strep, infant sex, maternal weight gain, maternal age	272.270	.		
Step 1	1	Entered	Augmented labor	259.426	12.844	2	0.002
Step 2	2	Entered	Artificially ruptured membranes	249.393	10.033	1	0.002
Step 3	3	Entered	Induced labor	241.446	7.946	1	0.005

**Likelihood ratio tests**							
**Effect**				**Model fitting criteria**	**Likelihood ratio tests**		
				**−2 Log likelihood of reduced model**	**Chi-square**	**df**	**Sig.**
Intercept				241.446^c^	0.000	0	.
HDP				181.503	0.081	1	0.776
Beta-strep				246.719	5.273	2	0.072
Infant sex				244.502	3.055	1	0.080
Maternal weight gain (kg)				416.954	175.508	136	0.013
Maternal age				296.716	55.270	26	0.001
Induced labor				249.393	7.946	1	0.005
Augmented labor				251.464	10.018	2	0.007
Artificially ruptured membranes				255.018	13.572	1	0.000

Stepwise method: forward stepwise.

The chi-square statistic is the difference in −2 log-likelihoods between the final model and a reduced model. The reduced model is formed by omitting an effect from the final model. The null hypothesis is that all parameters of that effect are 0.

^a^ The chi-square for entry is based on the likelihood ratio test.

^b^ The chi-square for removal is based on the likelihood ratio test.

^c^ This reduced model is equivalent to the final model because omitting the effect does not increase the degrees of freedom.

HDP, hypertensive disorders of pregnancy.

Three neonatal deaths (3 per 1,018 participants) were reported with two deaths in NT mothers (2 per 832) and one death in the hypertensive group (1 per 186). Of the deaths in the NT group, one was an induced vaginal delivery of a stillborn infant born at 160 days (22 weeks, 6 days) and the second was a spontaneous vaginal delivery at 150 days (21 weeks, 3 days) of gestation. The HDP patient with neonatal death had a C-section for a non-reassuring fetal heart tracing at 202 days gestation (28 weeks, 6 days).

The risk of infant morbidity was evidenced by days of gestation and categorized as small (SGA) or large (LGA) gestational age at delivery. The minimum number of days of gestation was 150 (21 weeks, 1 day) and the maximum number of days of gestation for the cohort was 296 (42 weeks, 2 days). For the NT group, the minimum days of gestation was 150 (21 weeks, 1 day), and the maximum days of gestation was 296 (42 weeks, 2 days).

The NT and HDP groups were significantly different (*p* = 0.000) in terms of gestational age. When assessing the gestational period of each group of mothers, the HDP group had a low of 260 days (37 weeks, 1 day) gestation with a maximum gestational period of 281 (40 weeks, 1 day). For the CH group, the minimum was 244 days (34 weeks, 6 days) with a maximum of 285 days (40 weeks, 5 days) for the GH group, and the minimum was 202 days (37 weeks, 1 day) with a maximum of 288 days (41 weeks, 1 day) for the PREE group.

The rates of preterm and late preterm deliveries were 3% for NT and 3.2% for HDP mothers. A larger percentage of infants were delivered at full term in the HDP group (94.6%) as 16% of NT deliveries were late term, whereas only 2.2% of HDP delivered late term as shown in Table 3.

**Table 3.** Blood Pressure by Infant Birth Status Crosstabulation (*p* = 0.000)

Blood pressure	Birth status				Total
	Very preterm	Late preterm	Full term	Late term	
Normotensive	0.4%	2.6%	81%	16%	100%
Hypertensive	0.4%	2.5%	95%	2.1%	100%

**FIG. 3.**
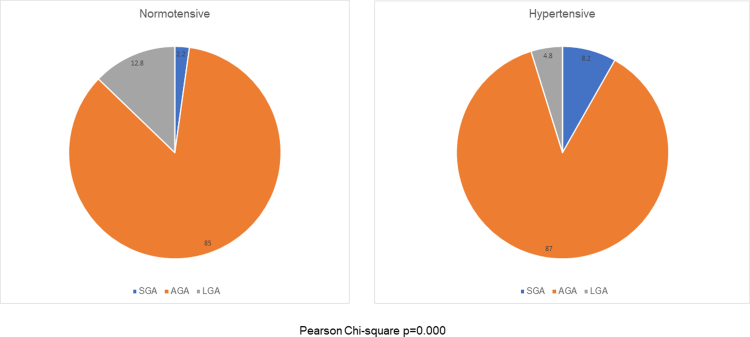
Infant birth weight status: SGA, AGA, and LGA. AGA, appropriate for gestational age; LGA, large for gestational age; SGA, small for gestational age.

Infant weight in the groups followed a normal distribution as shown in Figure 3. For the cohort, infant weight ranged from a low of 370 g to a high of 4,875 g with a group mean of 3,403 g. The overall SGA rate was 3.4%, with 85.2% born appropriate for gestational age and 11.4% LGA. When compared with NT and HDP, the NT group had an SGA rate of 2.3% compared to the HDP group at 8.2%, which was statistically significant (*p* = 0.001). The LGA rate in the HDP group was significantly lower (4.9%) than that in the NT group (12.8%; *p* = 0.001).

The mean infant weight was significantly different between the NT (3,458 g) and HDP groups (3,161 g *p* ≤ 0.00001). A total of 2.8% of infants were born below 2,500 g, with 1.8% in the NT group and 7.1% in the HDP group born below 2,500 g (SGA). When categorized by type of hypertension, 2.3% of the CH group, 4.6% of the GH group, and 11.8% of the PREE group infants were born below 2,500 g. Only the PREE group was significantly different with Pearson chi-square *p* = 0.000.

Based on birth certificate data for the time frame of this study, an evaluation of the variation in pregnancy-associated hypertension in the United States indicated that the prevalence of CH was 1.9%, GH was 6.5% with an overall rate of HDP of 8.6%,^28^ and a rate of 6.45% for PE.^29^ The data in this study indicate a rate of HDP that is almost double the rate reported based on the birth certificate data with 18.3% of participants having HDP with CH accounting for 4.3%, GH in 6.4% of the participants, and 7.7% with PREE. Of the 186 participants with HDP, 23.1% were categorized as CH, 35% as GH, and 42% as PREE.

## Discussion

While active-duty service women are prescreened for any major medical condition before joining the Armed Forces and are held to a high physical standard before pregnancy, it is uncertain whether the higher rate of HDP is due to a higher proportion of dependents who are not subjected to the same physical standards. Our data analysis was unable to differentiate between dependent and active-duty women. Nonetheless, dependent mothers have as much access to prenatal care as active-duty service women. Although higher than the Center for Disease Control and Prevention (CDC) statistics reported in 2018, the rates in this study are lower than have been reported recently.^30^

While no peripartum maternal death was reported in our cohort, three neonatal deaths (3/1,018 or 2.9/1,000) were reported. Given a sample of 1,018 births and an infant mortality rate in the United States of 5.79 per 1,000 births, our reported neonatal death rate of 3.4 per 1,000 births would be consistent with expected results. At 2.9/1,000, our study had a lower rate than the neonatal mortality rate in the same eastern region of the United States reported in 2018 to be 3.7/1,000 and the national rate of 3.8/1,000 for the same period.^28^

When examining maternal weight, we hypothesized that our data would parallel previous studies published on weight gain associations with HDP; however, it did not. Although there were differences in the mean weight gain between NT and HDP with HPD, which had significantly higher weight gain, the difference in gain was not predictive of HDP in the regression model. It is possible that HDP may cause weight gain due to extravasation of fluids into tissues, so causation versus correlation is particularly unclear in this relationship.

Our study had some limitations. Our sample consists of both active-duty military members and military dependents. This population, due to a portion of the sample being active duty, is less representative of the general population because of the pre-health screening done before joining the armed services as well as the physical fitness standards maintained by active-duty women. While there is prompt access to care for both active duty and dependents, the mean age of the participates was 23–29 years, which is a prime reproductive age with a low likelihood of health comorbidities compared to pregnant women of advanced maternal age who are more represented in the general population. Maternal weight gain was not a significant predictor of HDP in this cohort; however, a difference was observed between the groups in terms of mean weight gain.

Unfortunately, this study did not have prepregnancy weight data, timing of weight gain, or height; therefore, prepregnancy obesity could not be evaluated, nor were there any data on the trimester of weight gain. Although other studies have shown that higher pregnancy weight gain was associated with an increased risk of HDP, our data do not support this conclusion. Additional research is needed to better understand the relationship between prepregnancy weight and weight gain in relation to HDP and birth outcome.

Along with HDP, maternal morbidity was identified in this study using maternal variables of birth interventions AROM, IL, AL, and C-section delivery. The C-section rate for the cohort was 19.7%, which was much lower than the 32.6% C-section rate for the region during the study.^29^ The C-section rate for the HDP group was even lower with a total of 15% C-section rate across all types of HDP. When evaluated by type of HDP, the C-section rate for CH, GH, and PREE was 9.3%, 10.8%, and 21.8%, respectively. GH and PE are thought to be spectrum disorders in mothers diagnosed with GH, who are at risk of disease progression to PREE, which may have contributed to the higher rate of cesarean delivery in the PREE group. The higher rate of PREE found in this study is consistent with previous data and is representative of the general population.

The rate of preterm delivery was 9.6% in the region and 9.9% for the United States when this study was conducted.^31^ The rate of prematurity in the cohort as a whole was lower for both NT at 3% and for HDP mothers at 3.2% in this study. This was higher than that reported in a study conducted at a joint military hospital out of the United States, with a prematurity rate of 2.5% of births in the cohort.^32^ Although higher than the previous military facility study, the significant preterm delivery discrepancy between military and civilian hospitals warrants further research.

In trying to minimize evaluation bias, these data can be compared to similar populations with similar access to health care, such as the military population. However, our data showed a higher HDP rate than that currently reported in the literature. A study by Nash et al. comparing active-duty military women who were deployed and not deployed for their first pregnancy, confirmed a historically lower HDP in their population^33^ than in our study. There are no studies currently available comparing data from active personnel to dependents, but all data reviewed are consistent with the increasing prevalence of HDP reported across the spectrum of the general populace.^24,34,35^

Low birth weight, defined as infants born weighing <2,500 g, for the same period in the region was 8.1% and 8.2% in the United States as a whole. In this cohort, the rate of infants born under 2,500 g was 2.8%, which was much lower than either the state or the national figures. In a comparable military hospital study conducted in 2018, the reported rate of low birth weight for their cohort was 2.4% (62/2,599), which is much lower than the rate reported for the region and the United States and slightly lower than the rate reported by Baig and Jamal.^32^

Data from this study confirm that the incidence of HDP has been increasing. The rate of HDP in this cohort was 18.3%, which was similar to the 19% rate reported for East Texas,^30^ but higher than the rates reported for other states.^36^ It has been reported in the international medical literature that the incidence of HDP has increased by up to 10.92% between 1990 and 2019.^2^ The evidence indicating that HDP is increasing in prevalence was further verified by this study.

## Conclusion

Morbidity associated with HDP in both mothers and infants is a major public health concern. Long-term complications of HDP have a significant impact on the health care system, with most of the high costs driven by infant health care costs associated with lower gestational age and greater adverse outcomes.^37^

In this study, maternal weight gain was not associated with HDP diagnosis. However, given the sample population inclusion of active-duty personnel and the mean age of 27.6 years with few low extremes of age, further research would be instrumental in determining the causative versus correlative role of timing in maternal weight gain compared to the role of prepregnancy weight in the development of HDP. By examining the timing and weight gain by trimester and associating weight gain with a dietary profile, there could be key links between the ever-expanding role of nutrition in promoting positive health outcomes.

## Abbreviations Used

AGA: appropriate for gestational age
AL: augmented labor
AROM: artificial rupture of membranes
BMI: body mass index
C-section: cesarean section
CH: chronic hypertension
CSEC: cesarean section delivery
GH: gestational hypertension
HDP: hypertensive disorders of Pregnancy
IL: induced labor
LGA: large for gestational age
NT: normotensive
PE: preeclampsia
PREE: preeclampsia/eclampsia
SGA: small for gestational age
VAG: vaginal delivery
